# Trifocal diffractive intraocular lens implantation in patients after previous corneal refractive laser surgery for myopia

**DOI:** 10.1186/s12886-020-01556-0

**Published:** 2020-07-17

**Authors:** Qiu-Mei Li, Feng Wang, Zhe-Ming Wu, Zhen Liu, Chuan Zhan, Bing-Heng Chen, Jing Sima, Knut Stieger, Shao-Wei Li

**Affiliations:** 1 Beijing Aier-Intech Eye Hospital, Beijing, 100021 China; 2 Department of Ophthalmology, Aier School of Ophthalmology, Central South University, Changsha, 410083 China; 3 Department of Ophthalmology, Justus-Liebig-University, 35385 Giessen, Germany; 4 Guangzhou Aier Eye Hospital, Guangzhou, 510260 China; 5 Chongqing Aier Eye Hospital, Chongqing, 400020 China; 6 Wanzhou Aier Eye Hospital, Chongqing, 404000 China; 7 Shenzhen Aier Eye Hospital, Shenzhen, 518005 China

**Keywords:** Trifocal IOL implantation, Cataract, LASIK, PRK, Myopia, Aphakic refraction technique

## Abstract

**Background:**

With the difficulties in IOL power calculation and the potential side effects occurring postoperatively, multifocal IOL implantation after previous corneal refractive surgery are rarely reported especially for the trifocal IOL. Herein we report the clinical observation of trifocal IOL implantation in patients with previous myopia excimer laser correction. In this study, a multi-formula average method was performed for the IOLs power calculation to improve the accuracy. Visual and refractive outcomes were analyzed, and the subjective quality of patients’ life was evaluated by questionnaires survey.

**Methods:**

This retrospective case series included patients with previous myopia excimer laser correction who underwent femtosecond laser assisted phacoemulsification and trifocal IOL (AT LISA tri 839 MP) implantation. Follow-up was done at 1-day, 1-month and 3-month to assess the visual outcomes. Outcome measures were uncorrected distance, intermediate and near visual acuity (UDVA, UIVA, UNVA), manifest refraction, defocus curve, and subjective quality of vision.

**Results:**

Twenty-one Eyes from sixteen patients (14 eyes with previous laser in situ keratomileusis and 7 eyes with previous photorefractive keratectomy) were included. Mean postoperative spherical equivalent (SE) at 3-month was − 0.56 D ± 0.49 SD, wherein, 10 eyes (47.6%) were within ±0.50 D of the desired emmetropia and 19 eyes (90.5%) were within ±1.0 D. Mean monocular UDVA, UIVA and UNVA (logMAR) at last visit were 0.02 ± 0.07, 0.10 ± 0.10, and 0.15 ± 0.11 respectively. Three patients (19%) reported halos and glare in postoperative 3 months, two of them needed to use spectacles to improve the intermediate visual acuity. Fifteen patients (94%) reported a satisfaction score of ≥3.5 out of 4.0, without any difficulty in daily activity. Thirteen patients (81%) did not need spectacles at all distances, while the other 3 patients (19%) used spectacles for near-distance related visual activity. Mean composite score of the VF-14 questionnaire was 95.00 ± 7.29 out of 100.

**Conclusions:**

Trifocal IOL implantation after myopia excimer laser correction could restore good distance, intermediate visual acuity and acceptable near visual acuity, and provide accurate refractive outcomes as well as high spectacles independence rate.

## Background

Corneal refractive laser surgery that began in the 1990s has been well developed and widely applied in ametropia correction for millions of patients. Over time, these patients eventually become presbyopic or cataractous and are more demanding for the freedom from glasses [1]. To that end, a further refractive procedure is required such as multifocal intraocular lens (IOL) implantation. With the continuous renewal and improvement of surgical equipment and implantation material, multifocal IOL implantation after cataract surgery has been increasingly applied in clinical practice and effectively restored visual acuity, thus becoming an alternative of refractive procedure [2–7]. Recently, some studies have shown that the multifocal IOL implantation could be safely and efficiently used for patients who had previously undergone corneal refractive laser surgery [8–11]. Obviously, the modern cataract surgery combining an intraocular lens implantation gradually becomes a refractive procedure rather than a treatment only for blindness. However, due to the difficulties in IOL power calculation and the potential side effects occurring postoperatively such as glare or other visual acuity problems, only few reports of the clinical observation of multifocal IOL implantation after previous corneal refractive laser surgery exist, and even less for the trifocal IOL implantation.

Currently, bifocal IOLs are used as the major multifocal IOLs with only near and far focus and hence provide good distance and near visual acuity and moderate intermediate vision. As a newly emerging product from IOL multifocality technological development, trifocal IOLs were designed to provide three useful focal distances (near, intermediate and far) and thus improve the intermediate vision without impairing distance and near visual acuity [12–16]. An increasing number of reported clinical observations have demonstrated the positive outcome after trifocal IOL implantation and recent systematic reviews also showed the advantages of trifocal IOLs in comparison with bifocal IOLs [17, 18]. Within this trend, trifocal IOLs would become a reliable alternative option for cataract patients with previous corneal refractive laser surgery in the foreseeable future. So far, there are few studies reporting the visual outcomes of trifocal IOL implantation after previous corneal refractive surgery. Wang et al. recently reported on a trifocal IOL implantation in eyes with nuclear cataract about 15 years after myopic LASIK surgery, which restored vision efficiently [19]. Chow and co-worker thereafter reported a case series of trifocal IOL implantation for post-myopic LASIK patients providing a good visual outcome at both near and distance vision [20]. To further confirm the clinical effect of trifocal IOL implantation after previous corneal refractive surgery, we herein present a retrospective case series of trifocal IOL (AT LISA tri 839MP) implantation in eyes with previous corneal refractive laser surgery for myopia. In this study, a multi-formula average method was performed for the IOLs power calculation to improve the accuracy. The visual and refractive outcomes were analyzed, and patient satisfaction and subjective quality of vision were also evaluated using VF-14 questionnaire and another short questionnaire concerning negative visual symptoms.

## Methods

This retrospective case series included patients who had previously undergone LASIK or PRK for myopia and underwent femtosecond laser assisted phacoemulsification and trifocal IOL (AT LISA tri 839MP, Carl Zeiss Meditec AG, Jena, Germany) implantation in AIER Group’s Eye Hospitals (Beijing Aier-Intech Eye Hospital, Guangzhou Aier Eye Hospital, Chongqing Aier Eye Hospital, Wanzhou Aier Eye Hospital and Shenzhen Aier Eye Hospital) from January 2017 to May 2019. Ethics Committee approval by the Institutional Review Board of the Aier School of Ophthalmology of Central South University was obtained for the present study protocol that adhered to the tenets of the Declaration of Helsinki. Written informed consents were obtained from all the enrolled patients.

The inclusion criteria consisted of eyes (1) that had surgery indication for cataract surgery without any contraindications of ocular surgical therapy in the preoperative examination, (2) with corneal astigmatism ≤1.5 D and the angles of Kappa and Alpha both being < 0.3 mm, and (3) had no complications of posterior capsular rupture or zonular dialysis during the cataract surgery. The exclusion criteria included eyes that (1) had decentered ablation (decentration > 0.5 mm), corneal scar, retinoschisis, haze, myopic retinopathy, retinoschisis or retinal detachment after myopia excimer laser correction, (2) with corneal astigmatism ≥1.5 D or irregular astigmatism ≥0.3 μm, and (3) had inflammation, glaucoma or other diseases that might affect the multifocal IOL implantation.

### Patient examinations

Enrolled patients all underwent preoperative ophthalmologic examinations including corrected distance visual acuity (CDVA), UDVA, manifest refraction, retinal visual acuity, intraocular pressure, ocular A and B-scan ultrasonography, non-contact specular microscope, optical coherence tomography (OCT) and ray tracing aberrometry. The retinal visual acuity was assessed with Lambda 100 retinometer (Heine, Germany). The axial length, keratometry and anterior chamber depth were measured using the LenStar LS900 (Haag Streit, Switzerland).

The postoperative measurements at 1-day, 1-month and 3-month included CDVA, UDVA, UIVA, UNVA, and the subjective manifest refractions (spherical equivalent) as well as their changes. Through-focus monocular logMAR acuity (defocus curve) was also measured. The spectacle independence rate, satisfaction, and visual symptoms were recorded at 3-month postoperatively with the Visual Function index 14 (VF-14) questionnaire, which provides an index of functional impairment in patients with cataract [21, 22], as well as another short questionnaire concerning negative visual symptoms. VF-14 was translated into Chinese [23] and based on 14 uncorrected vision–dependent daily activities, scoring each item with regard to the degree of difficulty as follows: no difficulty (4 score), a little difficulty (3 score), moderate difficulty (2 score), quite difficult (1 score), or impossible to perform the task (0 score). Items were not included in the scoring if patients could not perform the activity for reasons other than vision-related. Scores on all activities were averaged, and the mean score was then multiplied by 25. The resulting VF-14 score ranged from 0 (worst functional impairment) to 100 (no disability) [24]. The short questionnaire with regard to some negative visual symptoms commonly observed after cataract surgery, such as halo, glare, was recorded by the correspondent surgeon.

### Surgical technique

All the surgeries were performed combining femtosecond laser-assisted phacoemulsification and intraocular lens implantation by experienced surgeons. For the eyes with corneal astigmatism between 0.75 D and 1.5 D (6 eyes), femtosecond laser-assisted corneal relaxing incision was performed for correction. Preoperatively, the surgeons used tropicamide to maintain pupil dilation intraoperatively. Under topical anaesthesia, capsulotomies (diameters were all set as 5.2 mm), lens fragmentation and corneal relaxing incisions were performed subsequently using the LenSx femtosecond laser (Alcon Laboratories, Inc., Fort Worth, Texas, USA). Phacoemulsification was then performed using standard ultrasound technique. In case of the patients with high myopia who underwent femtosecond laser surgery, both anterior and posterior capsules were thoroughly polished to reduce the risk of capsule contraction. The trifocal IOL (AT LISA tri 839MP) was implanted in the capsular bag using an injector. The residual ophthalmic viscosurgical device was removed, and the position of the lens was adjusted. All incisions were hydrated and the patients’ conjunctival sac was treated with dexamethasone-tobramycin ophthalmic ointment.

For lens power calculation, a multi-formula average method was performed, in which 4 formulas of Hagis-L [25], Barrett True K [26], Shammas No-History [27] and ray-tracing methods [28] setting the target refraction as postoperative emmetropia were used. The implanted IOL power was the average of the calculated results from all 4 formulas. For the first treated eye of patients who underwent bilateral implantation and the eyes with a large difference (> 1.0 D) in the IOL power calculations using the 4 formulas, a modified aphakic refraction technique was applied based on the reported aphakic refraction procedure by Dr. Mackool [29]. At first, the cataract removal was performed without IOL implantation. 1 Day later, manifest refraction examination was performed for the lens power calculation followed by the IOL insertion. All the two-staged-procedure surgeries were performed in the same fashion after obtaining patient’s consent. After 1 week, another eye surgery would be performed.

Postoperative treatment included one drop of tobramycin-dexamethasone eye drops every 2 h for 3 days, afterward four times per day until 2 weeks, and then three times per day for another 2 weeks; and one drop of levofloxacin eye drops four times every day for 1 week; pranoprofen and sodium hyaluronate eye drops were administered as appropriate.

### Statistical analysis

Statistical analysis was performed using Microsoft Excel. Mean (± SD) was reported for continuous variables. Normal probability plots and Kolmogorov-Smirnov and Shapiro-Wilk tests were used to check the normality of data in SPSS software. Pre- and post-UDVA, pre- and post-CDVA outcomes were compared with the t test. Nonparametric tests were used to evaluate differences within groups. Differences were considered statistically significant when the P value was less than 0.05.

## Results

This study comprised 21 eyes of 16 patients (five men, 31.3%). The demographics of the study population are summarized in Table 1. The mean patient age at the time of IOL implantation was 48.5 ± 8.9 years (range, 36 to 66 years). The mean time gab between the refractive surgery and cataract surgery is 13.2 ± 4.3 years. Eleven (68.7%) and five patients (31.3%) underwent unilateral and bilateral implantation respectively. No complications developed intraoperatively. No further laser enhancement was performed. The mean logMAR of UDVA and CDVA were 0.83 ± 0.48 (range, 0.22 to 1.70) and 0.37 ± 0.30 (range, 0.00 to 1.00), respectively. The mean axial length was 27.70 mm ± 2.20 (range, 25.13 to 33.00 mm). The mean preoperative spherical equivalent (SE) was − 5.49 D ± 5.75 (range, − 18.75 to + 1.37 D).

**Table 1:** Demographics of the patients before IOL implantation

Item	Number or Mean ± SD (range)
Number of eyes (N)	21
Number of patients (n)	16
Sex (male/female, n/n)	5/11
Years after refractive surgery	13.2 ± 4.3 (3, 21)
*Refractive surgery procedures (numbers of eyes), (percentage)*	
PRK	7 (33.3%)
LASIK	14 (66.7%)
*Symptoms (numbers of patients), (percentage)*	
Bilateral	5 (31.3%)
Unilateral	11 (68.7%)
Eyes with modified aphakic refraction technique	11 (52.4%)
Mean age (Years) ± SD (range)	48.5 ± 8.9 (36, 66)
Mean *pre*-UDVA (logMAR) ± SD (range)	0.83 ± 0.48 (0.22, 1.70)
Mean *pre*-CDVA (logMAR) ± SD (range)	0.37 ± 0.30 (0.00, 1.00)
Mean *pre*-SE (D) ± SD (range)	−5.49 ± 5.75 (−18.75, + 1.37)
Mean axial length (mm) ± SD (range)	27.70 ± 2.20 (25.13, 33.00)
Mean keratometry (D) ± SD (range)	38.36 ± 2.27 (34.34, 41.94)
Mean power implanted IOL (D) ± SD (range)	17.64 ± 3.87 (8.0, 23.00)

*IOL* intraocular lens, *LASIK* laser in situ keratomileusis, *PRK* photorefractive keratectomy, *UDVA* uncorrected distance visual acuity, *SE* spherical equivalent, *pre-* preoperative

### Refraction

**Fig. 1:**
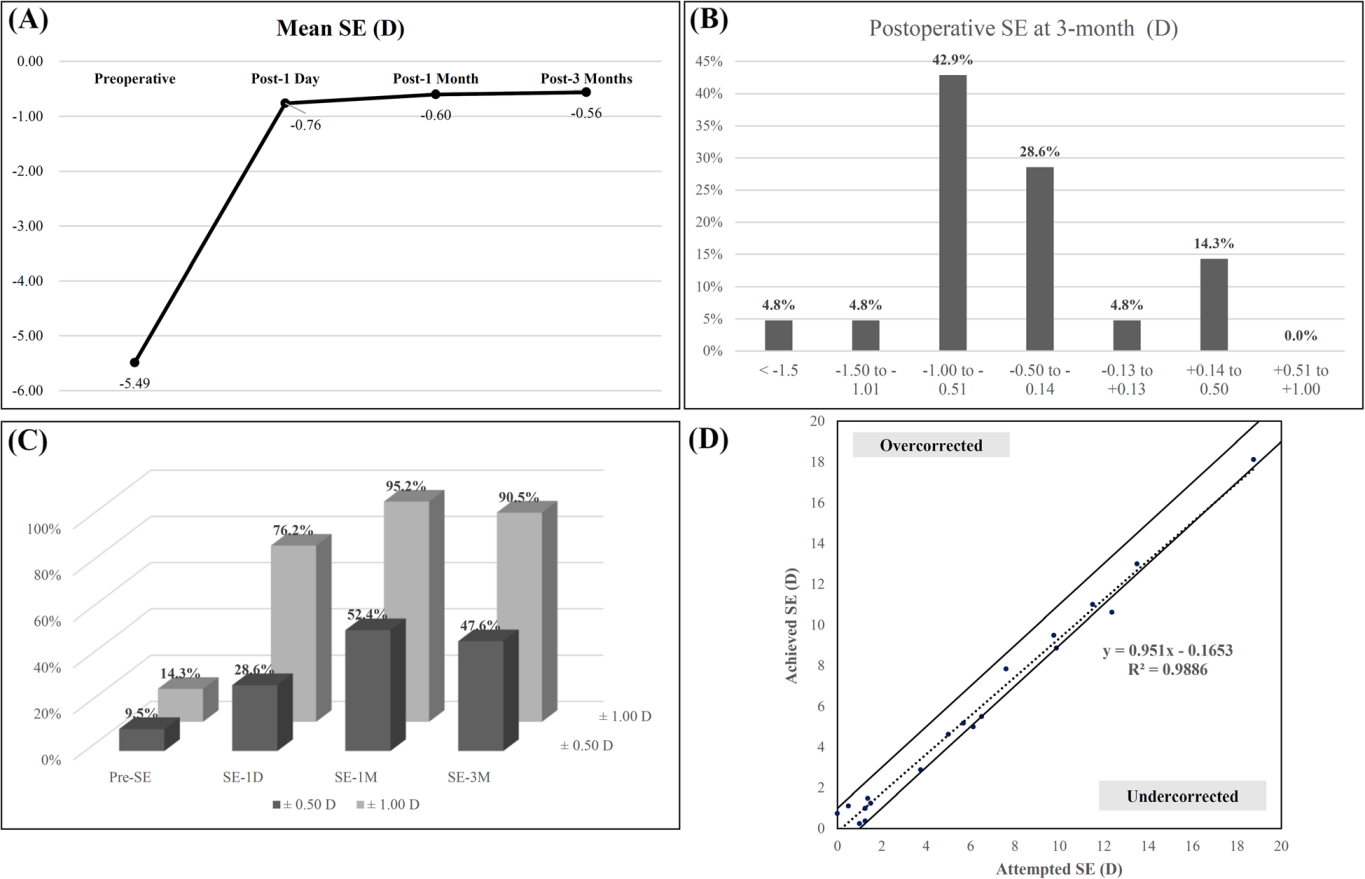
Refraction outcomes. **a** Changes of mean SE at the three follow-up intervals (1-day, 1-month and 3-month); **b** Histograms of the postoperative refractive accuracy; **c** Percentages of eyes within ±0.5 D and ± 1.0 D of emmetropia; **d** Spherical equivalent attempted versus achieved

After lens extraction and IOL implantation at 3-month, the mean SE was reduced to − 0.56 D ± 0.49. Figure 1a shows the change of mean SE at the three follow-up intervals (1-day, 1-month and 3-month). The histograms of the postoperative refractive accuracy are presented in Fig. 1b. Ten eyes (47.6%) were within ±0.50 D of the desired emmetropia and 19 eyes (90.5%) within ±1.0 D at postoperative 3 months. In contrast, only 3 eyes (14.3%) were within ±1.0 D preoperatively (Fig. 1c). Figure 1d shows the attempted versus achieved SE refraction. At 3-month follow-up, the overall rate of overcorrection was 0%, while the overall rate of undercorrection was 9.5% (2 eyes).

### Visual acuity

**Fig. 2:**
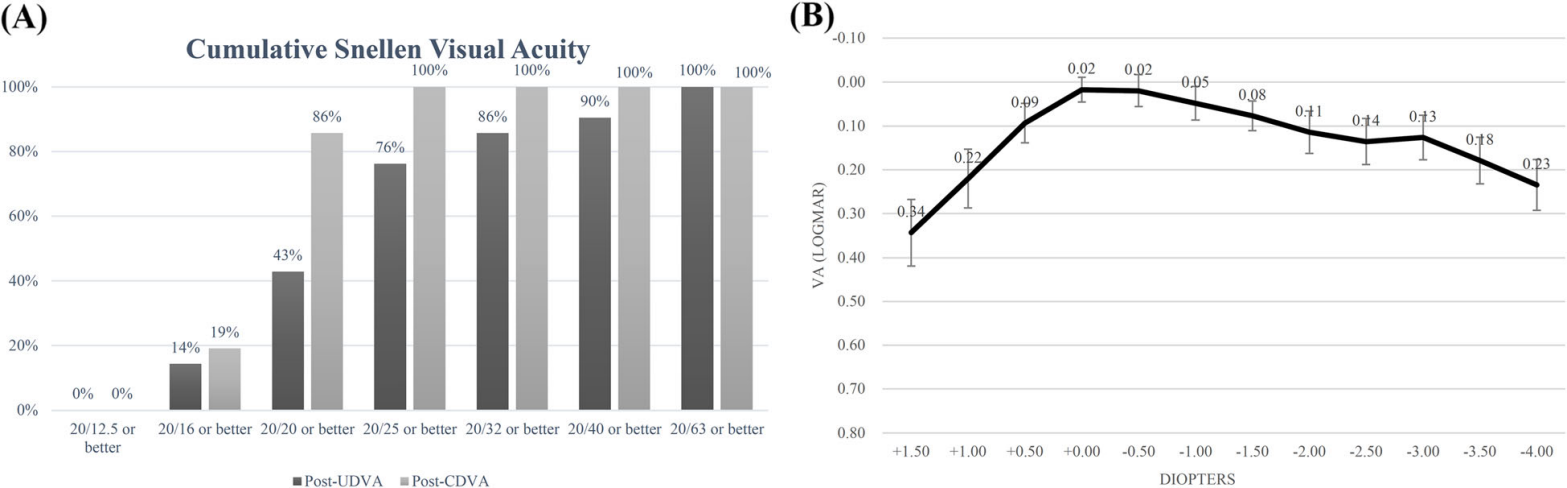
Visual outcomes. **a** Postoperative cumulative Snellen visual acuity; **b** Monocular distance-corrected defocus curve given in logMAR at 1-month postoperatively (*N* = 20)

The mean postoperative CDVA was 0.00 ± 0.05 logMAR, the mean postoperative UDVA was 0.10 ± 0.16 logMAR at the first day postoperatively (Table 2). All the surgery eyes (21 eyes, 100%) achieved postoperative CDVA ≥20/25, while the percentage for UDVA ≥20/25 was 76% (for the postoperative cumulative Snellen visual acuity, see Fig. 2a). Additionally, the mean postoperative UNVA, UIVA and UDVA at 3-month were 0.15 ± 0.11 logMAR, 0.10 ± 0.10 logMAR and 0.02 ± 0.07 logMAR respectively. All obtained monocular visual acuity data at the last visit are summarized in Table 2.

**Table 2:** Monocular visual acuity (logMAR)

Parameter	Preoperative		Postoperative at 1-day		***P***	Postoperative at 3-months	
	Mean ± SD	Range	Mean ± SD	Range		Mean ± SD	Range
**CDVA**	0.37 ± 0.30	0.00, 1.00	0.00 ± 0.05	−0.08, 0.10	< 0.001	\	\
**UDVA**	0.83 ± 0.48	0.22, 1.70	0.10 ± 0.16	−0.08, 0.52	< 0.001	0.02 ± 0.07	−0.08, 0.22
**UIVA**	\	\	\	\	\	0.10 ± 0.10	0.00, 0.30
**UNVA**	\	\	\	\	\	0.15 ± 0.11	0.00, 0.40

### Defocus curve

Monocular defocus curve at 1-month (Fig. 2b, results of 20 eyes) showed best visual acuity (0.02 logMAR and 0.13 logMAR) with defocus of 0.00 D and − 3.00 D, simulating distances of 4 m and 33 cm. In the range between the peaks, the curve transited smoothly and reached the bottom as 0.14 logMAR at − 2.50 D. Besides the defocus of 1.50 D (with a visual acuity of 0.34 logMAR), all visual acuities tested at other defocus diopters were better than 0.23 logMAR, maintaining a functional range of visual acuity.

### Questionnaires

Tables 3 and 4 summarized the results of the VF-14 and supplementary questionnaires. Three patients (19%) reported halos giving the least mean scores of all items in VF-14 questionnaire as 3.42, 3.50 and 3.64 respectively, two of them needed to use spectacles to improve the intermediate visual acuity. Thirteen patients (81%) did not need spectacles at all distances, while the other three patients (19%) reported the need to use spectacles only for the near-distance related visual activities such as reading books and doing handwork. Fifteen patients (94%) reported a satisfaction score of ≥3.5 out of 4.0, without any difficulty in daily activity.

**Table 3:** Three-month postoperative responses to visual function questionnaire (VF-14) items^a^

Item	Score (mean) ± SD	Score multiplied by 25	Spectacle independence rate
Reading small print	3.06 ± 0.77	77	81%
Reading a newspaper or a book	3.63 ± 0.62	91	88%
Reading a large-print book or numbers on a telephone	3.88 ± 0.34	97	100%
Recognizing people when they are close to you	4.00 ± 0.00	100	100%
Seeing steps, stairs, or curbs	4.00 ± 0.00	100	100%
Reading traffic, street, or store signs	4.00 ± 0.00	100	100%
Doing fine handwork like sewing^b^	3.36 ± 0.50	84	87%
Writing checks or filling out forms	3.81 ± 0.40	95	100%
Playing games such as bingo, dominos, card games, mahjong	4.00 ± 0.00	100	100%
Taking part in sports like bowling, handball, tennis, golf	3.94 ± 0.25	98	100%
Cooking	3.94 ± 0.25	98	100%
Watching television	3.88 ± 0.34	97	100%
Driving during the day^‡^	4.00 ± 0.00	100	100%
Driving at night^‡^	3.50 ± 0.76	88	100%

^a^Score scale: 4, no difficulty; 3, a little difficulty; 2, moderate difficulty; 1, quite difficult; 0, impossible to perform; ^b^ N = 15; ^‡^ N = 8

**Table 4:** Results of the short questionnaire concerning negative visual symptoms in postoperative 3 months

Negative visual symptoms in postoperative 3 months	Number of patients	Percentage
Halos	3	19%
Glare	3	19%
Flare	0	0%

## Discussion

Previous studies of eyes after corneal refractive laser surgery have focused primarily on monofocal IOLs [26, 30–32] and bifocal IOLs [8–11]. In our study, we present this retrospective case series of trifocal IOL (AT LISA tri839MP) implantation in patients who underwent a previous corneal refractive laser surgery for myopia. The AT LISA tri839MP used in our study is a monolithic diffractive trifocal intraocular lens which is made of collapsible hydrophilic acrylate (25%) with a hydrophobic surface. Its optical zone has a diameter of 6.0 mm, a total diameter of 11.0 mm, and a four-turn intraocular lens with a zero angle. The intraocular lens combines a bifocal diffraction range from 4.34 to 6.00 mm, and a trifocal diffraction range (over 4.34 mm) on the front surface of the lens, which is achieved based on the asymmetrical light split, that is 30, 20, and 50% of the incoming light is split at near focus, intermediate focus, and distant focus respectively. It was developed to overcome the photic phenomena and the poor level of intermediate vision of traditional multifocal IOLs [33] and has already been demonstrated to have good outcomes of visual acuity at near, intermediate and far distance and high postoperative satisfaction [34]. Patients were extensively counselled regarding the possible side effects of the treatment such as night glare, halos and decreased contrast sensitivity, even the possibility of deteriorated visual quality after a refractive corneal laser procedure. The final decision was made based on their wishes to restore vision and hence becoming independent of spectacles.

Intraocular lens implantation in eyes with previous corneal refractive laser surgery is full of challenges due to the difficulty in IOL power calculation [30, 35, 36]. To date, some reported IOL power calculation methods have been used for IOL implantation in eyes with previous corneal refractive laser surgery providing relatively accurate outcomes [31, 37, 38]. However, obvious hyperopia is often observed postoperatively [39, 40]. For the lens power calculation in the present study, a multi-formula average method was applied which has been described and proven to be accurate in our previous study [41]. Additionally, corneal edema and intraocular pressure (IOP) changes could also affect the intraoperative calculation of lens power. A modified aphakic refraction technique was used in our study for the first operative eyes of patients who underwent bilateral implantation and the eyes with large difference (> 1.0 D) in the IOL calculated results from multi-formulas. The time gap between the cataract removal and manifest aphakic refraction was extended from 30 min (Dr. Mackool’s procedure [29]) to 1 day. This extension has been demonstrated to increase the stabilization of refraction in our previous observation, and hence reduce the error giving a more accurate calculation result. Both these improvements were made based on the previous investigation and to pursuit accuracy in IOL power calculation.

In the refraction outcome, the mean postoperative SE was reduced to − 0.56 D ± 0.49 while Chow’s study reported a − 0.92 D ± 0.76 with a significant myopic shift [20]. In addition, 10 eyes (47.6%) were within ±0.50 D of the desired emmetropia and 19 eyes (90.5%) within ±1.0 D at postoperative 3 months in our study. All our cases were targeted for emmetropia, and the results were similar to Vrijman’s study with a lower percentage of eyes with ±0.50 D. This is because most of the patients included were preoperatively high myopia with either less spherical equivalent (9 eyes, 43%, with < − 6.00 D) or longer axial length (16 eyes, 76%, with > 26.00 mm). It is consistent with Vrijman’s finding that results were less predictable in eyes with myopia greater than 6.0 D [10].

The results of vision outcomes show a statically significant improvement after surgery. The mean postoperative CDVA and UDVA was 0.00 ± 0.05 and 0.10 ± 0.16 (logMAR), respectively. Both these two outcomes are significantly better than preoperative outcomes (Table 2, P < 0.001). The mean value of postoperative CDVA agrees with the result of Chang and co-workers (20/19, Snellen Vision) by converting into Snellen Vision [11]. To accurately evaluate the visual outcomes of trifocal IOLs after previous corneal refractive surgery, the monocular UDVA, UIVA and UNVA were recorded at last follow up in our study, and the mean values (logMAR) were 0.02 ± 0.07, 0.10 ± 0.10 and 0.15 ± 0.11 respectively. However, among the few reports using multifocal IOL implantation, only Chang’s study recorded the same visual outcomes wherein the mean UIVA was 0.22 ± 0.15 (logMAR) as higher than the value we observed. These results indicated that the trifocal IOL implantation for the cataract patients who previously had underwent myopia excimer laser correction, could restore good distance, intermediate visual acuity and acceptable near visual acuity.

As an important indicator for the vision over the entire range, defocus curve was also recorded in this study. In 2015, Jonker et al. compared the defocus curves after trifocal IOL implantation with that after bifocal IOL implantation in cataract patients [13]. In their comparison, the defocus curves of the trifocal IOL group showed a more continuous performance at the intermediate range under photopic and mesopic conditions. In the defocus curve of our study, the curve transited smoothly in the range between the peaks with defocus of 0.00 D and − 3.00 D and reached the bottom as 0.14 logMAR at − 2.50 D. Besides the defocus of 1.50 D (with a mean visual acuity of 0.34 logMAR), all visual acuities tested at other defocus diopters were better than 0.23 logMAR, maintaining a functional range of visual acuity. These results further demonstrate the good and wide range visual outcomes of trifocal IOL implantation after previous corneal refractive laser surgery for myopia.

In terms of the questionnaires survey, three patients (19%) reported halos and glare in postoperative 3 months, while no patient reported flare. Two of these three patients needed to use spectacles to read small print or a newspaper or book or to do some handwork. Looking back on the preoperative parameters of the two patients, both of their surgery eyes were with high myopia (preoperative spherical equivalents are − 7.60 and − 12.37 respectively). It seems that the capsule of high myopic patients is larger, and the stability of intraocular lenses is slightly worse compared to normal patients. The position and functional deviation of intraocular lens might lead to bad visual symptoms such as halos. In the other hand, the preoperative symptoms of halos or glare, and the status of the fellow untreated eye from patients who was performed unilateral surgery, might affect the postoperative visual outcomes and interfere with the clinical analysis of the efficiency of trifocal IOL implantation. Unfortunately, all the information had not been recorded preoperatively. Nevertheless, the total spectacles independence rate is 81% (13 patients), and those patients without halos or glare all have scores better than 3.67. Overall, the questionnaires survey showed good quality of life postoperatively in the current study.

The limitation of this study was the small sample size. Undoubtedly, a larger sample size would be more helpful to shed light onto the efficiency of trifocal IOL implantation after previous corneal refractive laser surgery for myopia. However, due to the uncertain side effects of multifocal IOL implantation after previous corneal surgery, the number of patients who balanced the side effects by their demands for becoming independent of spectacles are rare. Nevertheless, the trifocal IOL implantation should be a good option to restore wide range visual acuity well for the cataract patients with previous corneal refractive laser surgery for myopia.

## Conclusions

In conclusion, the trifocal intraocular lens can safely provide a full range of adequate vision and accurate refractive outcomes for cataract patients after myopic excimer laser correction. The high spectacle independence rate affords the patients a high satisfaction. Only few side effects such as halos and glare were observed and are possibly caused by extremely high myopia in few of the patients.

## Data Availability

The analytic data used to support the findings of this study are included within the article. Other original data used to support the findings of this study are available from the corresponding author upon request.

## Abbreviations

IOL: Intraocular lens
UDVA: Uncorrected distance visual acuity
UIVA: Uncorrected intermediate visual acuity
UNVA: Uncorrected near visual acuity
LASIK: Laser in situ keratomileusis
PRK: Photorefractive keratectomy
SE: Spherical equivalent
D: Diopter
SD: Standard deviation
logMAR: Logarithm of the minimal angle of resolution
CDVA: Corrected distance visual acuity
OCT: Optical coherence tomography
IOP: Intraocular pressure

## References

[1] Iijima K, Kamiya K, Shimizu K, Igarashi A, Komatsu M (2015). Demographics of patients having cataract surgery after laser in situ keratomileusis. J Cataract Refract Surg.

[2] Yoshino M, Bissen-Miyajima H, Minami K, Taira Y (2013). Five-year postoperative outcomes of apodized diffractive multifocal intraocular lens implantation. Jpn J Ophthalmol.

[3] van der Linden JW, van der Meulen IJ, Mourits MP, Lapid-Gortzak R (2013). Comparison of a hydrophilic and a hydrophobic apodized diffractive multifocal intraocular lens. Int Ophthalmol.

[4] de Vries NE, Webers CA, Montes-Mico R, Tahzib NG, Cheng YY, de Brabander J, Hendrikse F, Nuijts RM (2008). Long-term follow-up of a multifocal apodized diffractive intraocular lens after cataract surgery. J Cataract Refract Surg.

[5] Rosen E, Alio JL, Dick HB, Dell S, Slade S (2016). Efficacy and safety of multifocal intraocular lenses following cataract and refractive lens exchange: Metaanalysis of peer-reviewed publications. J Cataract Refract Surg.

[6] Liu X, Xie L, Huang Y (2018). Comparison of the visual performance after implantation of bifocal and trifocal intraocular lenses having an identical platform. J Refract Surg.

[7] Li S, Jie Y (2019). Cataract surgery and lens implantation. Curr Opin Ophthalmol.

[8] Fernandez-Vega L, Madrid-Costa D, Alfonso JF, Montes-Mico R, Poo-Lopez A (2009). Optical and visual performance of diffractive intraocular lens implantation after myopic laser in situ keratomileusis. J Cataract Refract Surg.

[9] Muftuoglu O, Dao L, Mootha VV, Verity SM, Bowman RW, Cavanagh HD, McCulley JP (2010). Apodized diffractive intraocular lens implantation after laser in situ keratomileusis with or without subsequent excimer laser enhancement. J Cataract Refract Surg.

[10] Vrijman V, van der Linden JW, van der Meulen IJE, Mourits MP, Lapid-Gortzak R (2017). Multifocal intraocular lens implantation after previous corneal refractive laser surgery for myopia. J Cataract Refract Surg.

[11] Chang JS, Ng JC, Chan VK, Law AK (2017). Visual outcomes, quality of vision, and quality of life of diffractive multifocal intraocular lens implantation after myopic laser in situ keratomileusis: a prospective, observational case series. J Ophthalmol.

[12] Vryghem JC, Heireman S (2013). Visual performance after the implantation of a new trifocal intraocular lens. Clin Ophthalmol.

[13] Jonker SM, Bauer NJ, Makhotkina NY, Berendschot TT, van den Biggelaar FJ, Nuijts RM (2015). Comparison of a trifocal intraocular lens with a +3.0 D bifocal IOL: results of a prospective randomized clinical trial. J Cataract Refract Surg.

[14] Mojzis P, Kukuckova L, Majerova K, Liehneova K, Pinero DP (2014). Comparative analysis of the visual performance after cataract surgery with implantation of a bifocal or trifocal diffractive IOL. J Refract Surg.

[15] Plaza-Puche AB, Alio JL (2016). Analysis of defocus curves of different modern multifocal intraocular lenses. Eur J Ophthalmol.

[16] Plaza-Puche AB, Alio JL, Sala E, Mojzis P (2016). Impact of low mesopic contrast sensitivity outcomes in different types of modern multifocal intraocular lenses. Eur J Ophthalmol.

[17] Shen Z, Lin Y, Zhu Y, Liu X, Yan J, Yao K (2017). Clinical comparison of patient outcomes following implantation of trifocal or bifocal intraocular lenses: a systematic review and meta-analysis. Sci Rep.

[18] Xu ZQ, Cao DM, Chen X, Wu S, Wang X, Wu Q (2017). Comparison of clinical performance between trifocal and bifocal intraocular lenses: a meta-analysis. PLoS One.

[19] Wang W, Ni S, Li X, Chen X, Zhu Y, Xu W (2018). Femtosecond laser-assisted cataract surgery with implantation of a diffractive trifocal intraocular lens after laser in situ keratomileusis: a case report. BMC Ophthalmol.

[20] Chow SSW, Chan TCY, Ng ALK, Kwok AKH (2019). Outcomes of presbyopia-correcting intraocular lenses after laser in situ keratomileusis. Int Ophthalmol.

[21] Steinberg EP, Tielsch JM, Schein OD, Javitt JC, Sharkey P, Cassard SD, Legro MW, Diener-West M, Bass EB, Damiano AM (1994). The VF-14. An index of functional impairment in patients with cataract. Arch Ophthalmol.

[22] Alonso J, Espallargues M, Andersen TF, Cassard SD, Dunn E, Bernth-Petersen P, Norregaard JC, Black C, Steinberg EP, Anderson GF (1997). International applicability of the VF-14. An index of visual function in patients with cataracts. Ophthalmology.

[23] Khadka J, Huang J, Mollazadegan K, Gao R, Chen H, Zhang S, Wang Q, Pesudovs K (2014). Translation, cultural adaptation, and Rasch analysis of the visual function (VF-14) questionnaire. Invest Ophthalmol Vis Sci.

[24] Song X, Liu X, Wang W, Zhu Y, Qin Z, Lyu D, Shentu X, Xv W, Chen P, Ke Y (2020). Visual outcome and optical quality after implantation of zonal refractive multifocal and extended-range-of-vision IOLs: a prospective comparison. J Cataract Refract Surg.

[25] Haigis W (2008). Intraocular lens calculation after refractive surgery for myopia: Haigis-L formula. J Cataract Refract Surg.

[26] Abulafia A, Hill WE, Koch DD, Wang L, Barrett GD (2016). Accuracy of the Barrett true-K formula for intraocular lens power prediction after laser in situ keratomileusis or photorefractive keratectomy for myopia. J Cataract Refract Surg.

[27] Shammas HJ, Shammas MC (2007). No-history method of intraocular lens power calculation for cataract surgery after myopic laser in situ keratomileusis. J Cataract Refract Surg.

[28] Savini G, Bedei A, Barboni P, Ducoli P, Hoffer KJ (2014). Intraocular lens power calculation by ray-tracing after myopic excimer laser surgery. Am J Ophthalmol.

[29] Mackool RJ, Ko W, Mackool R (2006). Intraocular lens power calculation after laser in situ keratomileusis: Aphakic refraction technique. J Cataract Refract Surg.

[30] Wang L, Hill WE, Koch DD (2010). Evaluation of intraocular lens power prediction methods using the American Society of Cataract and Refractive Surgeons Post-Keratorefractive Intraocular Lens Power Calculator. J Cataract Refract Surg.

[31] Wang L, Tang M, Huang D, Weikert MP, Koch DD (2015). Comparison of newer intraocular lens power calculation methods for eyes after corneal refractive surgery. Ophthalmology.

[32] McCarthy M, Gavanski GM, Paton KE, Holland SP (2011). Intraocular lens power calculations after myopic laser refractive surgery: a comparison of methods in 173 eyes. Ophthalmology.

[33] Mojzis P, Majerova K, Hrckova L, Pinero DP (2015). Implantation of a diffractive trifocal intraocular lens: one-year follow-up. J Cataract Refract Surg.

[34] Kohnen T, Titke C, Bohm M (2016). Trifocal intraocular lens implantation to treat visual demands in various distances following lens removal. Am J Ophthalmol.

[35] Seitz B, Langenbucher A, Nguyen NX, Kus MM, Kuchle M (1999). Underestimation of intraocular lens power for cataract surgery after myopic photorefractive keratectomy. Ophthalmology.

[36] Chan TC, Liu D, Yu M, Jhanji V (2015). Longitudinal evaluation of posterior corneal elevation after laser refractive surgery using swept-source optical coherence tomography. Ophthalmology.

[37] Chen X, Yuan F, Wu L (2016). Metaanalysis of intraocular lens power calculation after laser refractive surgery in myopic eyes. J Cataract Refract Surg.

[38] Savini G, Hoffer KJ (2018). Intraocular lens power calculation in eyes with previous corneal refractive surgery. Eye Vis (Lond).

[39] Saiki M, Negishi K, Kato N, Torii H, Dogru M, Tsubota K (2014). Ray tracing software for intraocular lens power calculation after corneal excimer laser surgery. Jpn J Ophthalmol.

[40] Hoffer KJ (2009). Intraocular lens power calculation after previous laser refractive surgery. J Cataract Refract Surg.

[41] Yu ZX, Li SW, Huo DM, Xu M, Shi S, Liu C, Zhao R (2019). Evaluation of the accuracy of Sirius ray-tracing method for IOL power calculation in post-corneal-refractive-surgery eyes. Ophthalmol CHN.

